# Primary health care delivery models in rural and remote Australia – a systematic review

**DOI:** 10.1186/1472-6963-8-276

**Published:** 2008-12-29

**Authors:** John Wakerman, John S Humphreys, Robert Wells, Pim Kuipers, Philip Entwistle, Judith Jones

**Affiliations:** 1 Centre for Remote Health, a joint Centre of Flinders University & Charles Darwin University, PO Box 4066, Alice Springs, Northern Territory, 0870, Australia; 2 Monash University School of Rural Health, PO Box 666, Bendigo, Victoria, 3550, Australia; 3 Menzies Centre for Health Policy and College of Medicine and Health Sciences, Australian National University, Building 131 Garran Rd, Acton, ACT, 0200, Australia

## Abstract

**Background:**

One third of all Australians live outside of its major cities. Access to health services and health outcomes are generally poorer in rural and remote areas relative to metropolitan areas. In order to improve access to services, many new programs and models of service delivery have been trialled since the first National Rural Health Strategy in 1994. Inadequate evaluation of these initiatives has resulted in failure to garner knowledge, which would facilitate the establishment of evidence-based service models, sustain and systematise them over time and facilitate transfer of successful programs. This is the first study to systematically review the available published literature describing innovative models of comprehensive primary health care (PHC) in rural and remote Australia since the development of the first National Rural Health Strategy (1993–2006). The study aimed to describe what health service models were reported to work, where they worked and why.

**Methods:**

A reference group of experts in rural health assisted in the development and implementation of the study. Peer-reviewed publications were identified from the relevant electronic databases. 'Grey' literature was identified pragmatically from works known to the researchers, reference lists and from relevant websites. Data were extracted and synthesised from papers meeting inclusion criteria.

**Results:**

A total of 5391 abstracts were reviewed. Data were extracted finally from 76 'rural' and 17 'remote' papers. Synthesis of extracted data resulted in a typology of models with five broad groupings: discrete services, integrated services, comprehensive PHC, outreach models and virtual outreach models. Different model types assume prominence with increasing remoteness and decreasing population density. Whilst different models suit different locations, a number of 'environmental enablers' and 'essential service requirements' are common across all model types.

**Conclusion:**

Synthesised data suggest that, moving away from Australian coastal population centres, sustainable models are able to address diseconomies of scale which result from large distances and small dispersed populations. Based on the service requirements and enablers derived from analysis of reported successful PHC service models, we have developed a conceptual framework that is particularly useful in underpinning the development of sustainable PHC models in rural and remote communities.

## Background

One third of Australia's population lives outside its major cities [1]. Of this non-metropolitan population, almost twenty percent is dispersed across more than 1,500 rural and remote communities with fewer than 5,000 residents. Collectively these communities have a population the size of Sydney, Australia's largest city. Almost three-quarters of these small communities lie in the rural and remote areas furthest from large population centres [2]. More than one-third of these small communities are losing population and experiencing economic hardship [3-5].

People living in small rural and remote communities of Australia face significant health disadvantage. Generally, mortality and illness levels increase with distance from major cities [1]. Moreover, these communities are characterised by higher hospitalization rates and higher prevalence of health risk factors compared with metropolitan areas [1,6,7]. These rural and remote communities are further disadvantaged by reduced access to primary health care (PHC) providers and health services (in part a function of health and medical workforce shortages), leading in turn to lower utilisation rates than in urban areas and consequent poorer health status for rural residents [1].

Often these isolated rural and remote communities are too small to support traditional models of health delivery locally, so residents must access care from larger urban centres. Unfortunately, access to health services provided in larger centres remains a problem for many residents of isolated settlements. In many cases, their inability to access health services when required results in health needs not being adequately met, lack of continuity of care and an absence of monitoring of the effectiveness of services in terms of health outcomes [1]. It is clear that 'models of care in rural and remote areas must differ from those in metropolitan communities, incorporating strategies to account for these problems' [8].

In order to address these access and service problems, specific measures targeting rural health featured in annual national government budgets from the early 1990s. In 1994 the Australian Health Ministers' Conference (AHMC) endorsed the first National Rural Health Strategy [9], which was renewed in 1999 with the release of '*Healthy Horizons*, a framework to guide the development of health programs and services in rural, regional and remote Australia'[6]. Since 1999 the Commonwealth has made two major budgetary commitments to rural health: in 2000 (*More Doctors-Better Services*) and 2004 (*Rural Health Strategy*) [10,11]. These initiatives constitute a series of workforce enhancement measures, principally targeting the medical workforce.

Policy-makers are under increasing pressure to strengthen the link between evidence, policy development and program implementation. Although numerous approaches and models of service delivery have been trialled in rural and remote areas since the first National Rural Health Strategy, inadequate evaluation of these initiatives has resulted in failure to garner knowledge, which would facilitate the establishment of evidence-based service models, sustain and systematise them over time and facilitate transfer of successful programs to other jurisdictions [12-14].

The objective of this research was therefore to systematically review the available published literature describing innovative models of comprehensive primary health care in rural and remote Australia since the development and publication of the first National Rural Health Strategy in order to identify what rural and remote primary health care models work well, where and why.

## Methods

Whilst systematic reviews of mixed qualitative and quantitative papers aimed at informing policy can be complex and do not always accord with a pure methodological approach, our experience shows how they can still be conducted rigorously and effectively within constraining circumstances [15]. This systematic review adopted some elements of a 'realist synthesis' approach in its engagement with policymakers, reliance on 'grey' literature and in the development of a theoretical framework to explain what health service models worked well, in which location they worked and why they worked [16]. To assist in the development and implementation of the study a Reference Group was formed, comprising eleven recognised experts in rural and remote health, health economics, consumer issues, evaluation, PHC service provision and government policy making. Two international health services researchers were included in the Reference Group.

This paper addresses two key aspects of the systematic review. What were the key remote and rural PHC models in Australia since the first National Rural Health Strategy, and what specific structural or financial issues did they address? Secondly, what are the characteristics of appropriate PHC service models for rural and remote Australia?

Peer-reviewed ('black') publications were identified from electronic databases: Medline, CINAHL, EBM Reviews, and AMED through the metadatabase OVID, APAIS-Health, ATSIhealth, H&S, Meditext and RURAL through the metadatabase INFORMIT, and EMBASE. The research questions and relevant search terms were developed iteratively, in consultation with the Reference Group, and refined during the literature search process. 'Grey' literature was identified pragmatically from works known to the researchers and Reference Group members, from reference lists and from web searches of government departments, workforce agencies, professional associations, universities and similar organisations. The search for and review of literature was divided across two research sites – one rural and one remote – based on familiarity with specific literature. (The full detailed search strategy is available in the funder's web version report ).

Table 1 shows the final inclusion/exclusion criteria which defined the scope and number of publications reviewed.

**Table 1:** Inclusion and exclusion criteria

**CRITERIA**	**INCLUSION**	**EXCLUSION**
**Time period**	• 1993–2005	

**Language**	• English	

**Place of study**	• Australia	

**Geographical delimitation**	• Rural or remote	• No relevance to rural or remote

**Aspect of health care**	• Comprehensive primary health care model or component thereof	• Secondary or tertiary health care (unless specifically articulated or supporting primary care)

**Objectives**		
1. What structural and financial issues are addressed?	• Identifies or addresses some specific structural or financial aspect of primary health service provision	• Problem description (not based on any evidence or intervention)
2. What are the barriers to and facilitators of success	• Identifies reasons for success or failure leading to models uptake or sustainability over time	• Descriptions of individual professional groups or activities (not models or systems)
3. Characteristics of appropriate models	• Some primary or secondary evidence base underpins research or statement	
4. Evidence-informed principles or guidelines	• Key structural and financial characteristics are explicitly identified, considered or evaluated	

**Other**		• Clinical intervention or trial
		• Education and training initiatives which do not inform a PHC service delivery model in a direct way.

**Figure 1:**
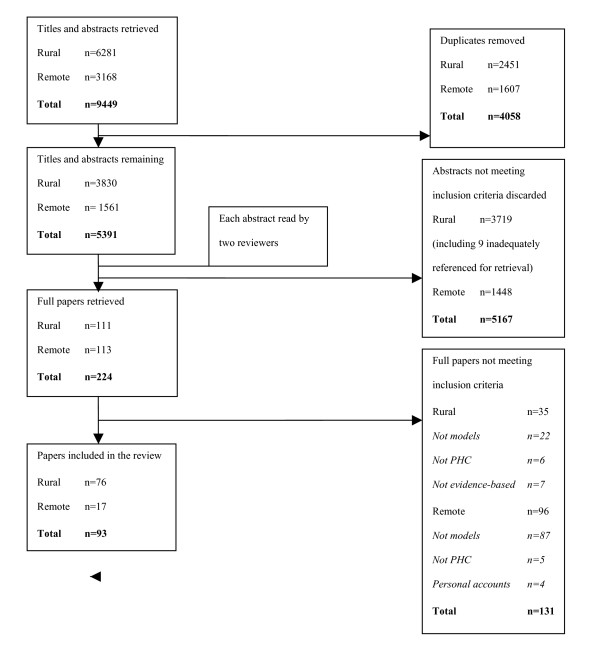
**Selection process for inclusion of papers in systematic review**.

Figure 1 summarises the selection process. Two reviewers independently read a total of 5,391 non-duplicate abstracts, comprising 3,830 'rural' and 1,561 'remote' abstracts. A sample comparison of 324 abstracts noted 80% concurrence between two readers. In instances where reviewers failed to reach initial agreement, a decision regarding selection was made on the basis of discussion and consensus.

As a result, 111 'rural' and 113 'remote' full papers were retrieved. Nine rural papers could not be retrieved due to inaccurate or incomplete citations. A further 35 'rural' papers and 96 'remote' papers were discarded as the full papers did not satisfy inclusion criteria. The remaining 76 rural papers and 17 remote papers were read and data extraction forms were completed. Data included location, service population size and model description. Data were assessed for quality and relevance. While quality was a consideration, relevance (as reflected in the inclusion and exclusion criteria) rather than quality was adopted as the principal decision criterion for inclusion.

A total of 59 items of 'grey' literature were also retrieved as full documents for 'rural' and 47 for 'remote'. Of the 'rural' documents, 49 dealt with models of service delivery. A further eight contained context-relevant information and two did not meet the inclusion criteria. Of the 47 'remote' documents, 19 met the inclusion criteria. Data were extracted using data extraction tables specifically developed for the systematic review. Of the total of 161 papers reviewed and analysed, only an indicative subset is referenced here. The full list is available in the funder's web version report (URL detailed above).

## Results

Synthesis of these data comprised progressive readings of each document, and the identification, categorisation and comparison of recurring themes across documents, involving the research team and the Reference Group. This allowed the development of a typology of models of PHC within five broad categories, each with a different rationale and addressing particular sentinel issues (see Table 2). Thirty six of the 161 papers constituted evaluations. Evaluation measures varied widely between studies. Some of these are summarised in Table 2.

**Table 2:** Typology of rural and remote PHC models

**CATEGORY**	**HEALTH SERVICE MODELS**	**RATIONALE/SENTINEL ISSUE**	**MEASURES OF SUCCESS**
**Discrete Services**	• 'Walk-in/Walk-out' (20)	***Sustainable ***medical ***workforce***(getting GPs into rural services)	• Increased number of doctors recruited (20)
	• Viable models/sustainable models (19, 21)		
	• University clinics (17, 18)		

**Integrated Services**	• Shared care (23, 24)	***Coordination ***between and ***access ***to services otherwise not available locally or not sufficient	• Decreased suicide rate; decreased GP isolation & increased confidence (23, 24)
	• Co-ordinated Care Trials (CCTs – mainstream) (25)		
	• PHC teams (multidisciplinary) (26–28)		• Decreased waiting times, reduced after hours call-outs; enhanced continuity of care; reduced inappropriate ED attendance (26)
	• Multi-Purpose Services Program (29–32)		• Increased service access; reduced residential care; increased home-based services (29–31)

**Comprehensive PHC Services**	• Aboriginal Controlled Community Health Services (including Aboriginal CCTs) (33–35, 36–38)	Primary focus on improved ***access ***to services	• Some improved processes of care (32); increased community participation (34); enhanced funding, improved community participation, improved governance, increased staff numbers, increased utilisation, new population health programs (37, 38)

**Outreach Services**	• Hub-and-spoke (40, 41)	***Access ***to service for communities too small to support discrete rural service. A secondary driver relates to sustainable workforce	• Increased occasions of service; increased workforce length of stay; increased referrals; improved cost-effectiveness (41)
	• Visiting/periodic services (42, 43)		
	• Fly-in, fly-out		

**Virtual Outreach Services (IT/Telehealth)**	• Virtual amalgamation (44, 45)	Use of IT to increase ***access ***to and ***sustain ***service for communities too small to support discrete rural service	• Improved access to records; reduced GP on call; increased consultation hours (44)
	• Virtual clinics – video pharmacy/assessment & monitoring		
	• Tele-health/-medicine		

Drawing on information about model type, location and service population size, it was evident that in general the different categories of models relate to different geographical contexts, with a notable association with population size and remoteness. While larger rural communities are generally able to support a greater variety of local, discrete, more specialised health care services, increasing remoteness and diminishing population size and density constrain service model options and increase the impetus for the development of more integrated and comprehensive primary health services in order to maximise the economies of scale and use of existing health workforce.

'*Discrete primary care services*' are delivered from an identifiable site located in the community they serve (for example, [17-22]). Their primary purpose is to sustain a general practitioner service in those rural and larger remote communities experiencing significant difficulties in recruiting and in retaining an adequate general practitioner workforce. They accomplish this through ensuring attractive practice opportunities for doctors and continuity of medical care for the community when doctors leave. Exemplars of this type are characterised by practice infrastructure owned and maintained by an entity such as a local council, university or other incorporated body, such that incoming general practitioners can execute both an 'easy entry' on recruitment and 'gracious exit' free of concern about return on capital investment.

'*Integrated services*' offer a range of integrated primary health care services from sites located in the communities they serve [23-32]. Their scope is significantly broader than general practitioner services, but may include coordination with general practitioner services. Integrated services provide single point access to a range of services and sufficient numbers of health professionals to ensure mutual professional support. Because these communities cannot usually sustain necessary allied health and specialist services in a discrete form, this model enables the population to sustain such a service.

'Integrated services', which usually emerge from a community health service or allied health team approach to primary health care services, comprise a variety of models. For example, the 'shared care' model of mental health care addresses access to and co-ordination of service across primary and specialist care [23,24]. The Multi Purpose Services (MPS) program provides a specific model of Commonwealth/state financing which allows for the co-location and common administration of acute care, residential aged care, community and allied health services, rehabilitation and health education activities [29-32].

'*Comprehensive primary health care services*' (CPHC) are best typified in Australia by the Aboriginal Community Controlled Health Services (ACCHSs). ACCHSs have adopted a primary health care approach to healthcare delivery over the past 30 years, and provide some of the best examples of this model [33-39]. CPHC services aim to improve health outcomes through better access to services and by addressing underlying social determinants of health. The main impetus for the development of ACCHSs has come from poor service access and availability, inadequate funding of services, low acceptance of mainstream services by Aboriginal patients, the poor health status of the Aboriginal population, and a desire for community control of these services. CPHC services are broader in scope than most 'Integrated Services' models. They include primary clinical care, preventive and health promotion activity, as well as education and development in relation to workforce training and governance/community capacity building.

'*Outreach models*' are characterised by the periodic supply of services from one location which has services to other locations which do not [40-43]. The arrangement may be either centrally located services providing services to satellite communities though a 'hub and spoke' arrangement, or some other visiting mechanism, such as where a general practitioner resident in one community may visit a second community for short periods, or services are supplied on a fly-in fly-out basis. Outreach services thus improve access to health services for widely dispersed and isolated populations and often co-exist with other integrated and comprehensive PHC services.

'*Telehealth*' and '*telemedicine*' have been widely used in Australia over the past decade as a means of overcoming problems of access to health care and the shortage of health professionals in rural and remote areas [44-49]. The extent to which telehealth and telemedicine constitute a 'model' of care in its own right is a moot point. In many cases, telemedicine and telehealth are used to augment other service delivery models.

## Discussion

This review represents the first comprehensive synthesis of published literature relating to Australian rural and remote models of PHC. The resultant typology of these models indicates that with increasing remoteness and decreasing population size and density, different model types assume prominence in addressing key PHC principles relating to accessibility, appropriateness and sustainability. The different models provide some guidance as to appropriate options for different settlement patterns in rural and remote areas. Where discrete general practice models can be sustained in sufficiently large country towns, alternative hub-and-spoke models may be required for delivering a full range of PHC services to smaller, more isolated communities. The need for ensuring that a comprehensive range of well-coordinated PHC services is locally accessible has become increasingly important as the prevalence of chronic disease grows with the ageing of Australia's rural and remote population. This typology is not prescriptive, nor are these models mutually exclusive, nor are they necessarily unique to rural and remote areas. Hence, for example, a hub-and-spoke model may share some aspect of shared care or similar collaborative arrangement, while a discrete GP model may provide an outreach service to outlying populations.

Underpinning all rural and remote models is Australia's ineluctable geography and demography. Beyond the coastal population centres, traditional models of health service provision have struggled to address diseconomies of scale which can result from large distances and small dispersed populations. Reportedly successful models, such as those that have emerged from this review, are able to aggregate a critical service population mass, whether it is a discrete town population or dispersed across a region. Evidence from the papers and discussion involving the Reference Group suggests that a critical minimum population base of about 5,000 inhabitants for rural regions and 2,000–3,000 people for remote communities is necessary to support a comprehensive and sustainable range of PHC services. The provision of PHC services to rural and remote communities smaller than these populations requires a model with characteristics that enable it to capture the necessary population aggregation required to support minimum service threshold requirements and thereby ensure adequate access to care.

Whilst there are different models that have been developed for different locations, a number of key environmental enablers and essential service requirements are common across the model types. Our synthesis has used the best available evidence to develop a conceptual framework that includes these service requirements and enablers for sustainable PHC services in small rural and remote communities (Tables 3 &4). This framework, endorsed and validated by the Reference Group, is particularly useful in underpinning the development of sustainable rural and remote PHC models.

**Table 3:** Essential service requirements and environmental enablers for PHC models in rural and remote communities

**CONTEXT Rural-Remote continuum**	**SERVICE OPTIONS**	**Environmental enablers**			**Essential service requirements**					
		Supportive policy	Common-wealth State relations	Community readiness	Work-force organis-ation	Work-force supply	Funding	Governance, management & leadership	Linkages	Infra-structure

**RURAL** (Characterised by larger, more closely settled communities)	**Discrete** eg: 'Easy Entry-Gracious Exit' model	The option for discrete primary health care services exists because community population catchments are sufficiently large to support them. The role of environmental enablers (while important) is less influential than in remote communities, and essential service requirements are more easily met even though supports are needed to address some aspects of services (such as workforce recruitment and retention).								
↓	**Integrated** eg: Multi-Purpose Services, Shared Care, Coordinated Care models	The need for service integration increases in order to maximise economies of scale and efficiencies in communities where individual services or competing services are not sustainable; single point of entry to the health system through locally available access pathways is important to co-ordinate patient care and reduce the need for patients to travel extensive distances; and maximise the range of locally available services.								
	**Comprehens-ive PHC** eg: Aboriginal Community Controlled Health Service model	This option ensures a comprehensive primary health care service is available in small, isolated, high-need communities where there are few, if any, alternative ways for delivering appropriate health care. The need to ensure that environmental enablers facilitate the delivery of appropriate care, minimise cost-shifting and duplication of activity and reporting, and maximise community participation in the service development are paramount. Flexibility in meeting essential service requirements is essential to take account of local needs and circumstances.								
**REMOTE** (Characterised by small populations dispersed over vast areas)	**Outreach/Virtual Outreach** eg: Hub and spoke; Fly-in, fly-out; Virtual clinics; Telehealth models	This option addresses the health needs of communities with populations too small to support permanent local services by providing access through virtual or periodic visiting services. Opportunities for community involvement and management will be more limited than with locally-based services, while co-ordination with any existing services is critical. Outreach models often co-exist with other model types- discrete, integrated and comprehensive PHC services.								

**Table 4:** Environmental enablers and essential service requirements for the 'Easy entry-gracious exit' discrete model

**Environmental enablers**	
Supportive policy	Initial Commonwealth grant funds enabled provision of practice equipment & furnished doctor housing. Following this, the Rural Medical Infrastructure Fund supported the model.

Commonwealth/State relations	Commonwealth and State agencies negotiated contracts of service to cash out some services, enabling a reliable income stream which enabled more specific income estimates for prospective doctors

Community readiness	There was a strong community commitment to finding solutions to the GP recruitment problem and local champions to drive the change to community ownership of infrastructure.

**Essential service requirements**	

Workforce	Recruits from a larger pool due to limited infrastructure investment requirement. Expanded GP role provides additional positions so can provide self-cover for after hours and on-call work.

Funding	Cashing out of hospital Visiting Medical Officer services, population health activity, Extended Primary Care (EPC) items, other Medicare and Retention Grants fund bulk-billing service.

Governance, management & leadership	Community, agencies (eg Division of General Practice, Area Health Service, Rural Workforce Agency) represented on Board. Professional business management instituted.

Linkages	Provides a platform for integration. Strong community & other linkages as above. Enables EPC activity involving allied health team.

Infrastructure	Community ownership through Rural Medical Infrastructure Fund, local government, Practice Incentives Program, Area Health Services. Potential collocation with hospital or community services.

Essential elements of sustainable PHC services for small rural and remote communities include a number of significant environmental *enablers *which are crucial in preparing an environment for change, together with a number of *essential requirements *that need to be met in order to improve access to PHC services. The enablers are: a supportive policy which ensures sustained service funding; co-ordination of policy and funding across national and state governments; and an appropriate level of community readiness for involvement in planning, implementation and monitoring of health service activity. The essential service requirements focus specifically on workforce – numbers and mix of staff; funding; governance, management and leadership; linkages, which include integration of services within an organisation and external linkages with other key organisations to ensure continuity of care; and infrastructure – physical infrastructure as well as adequate information and communication technology. These essential requirements are inter-related. Importantly, evidence from the systematic review showed that apparently successful examples of these models addressed the full range of essential requirements, with workforce, the focus of much current rural health policy in Australia, diminishing as such a critical barrier to sustainability. An example of how these factors apply to one discrete model is presented in Table 4.

This evidence-based framework provides principles or guidelines to guide the decisions of policy-makers in planning appropriate PHC service for small rural and remote communities [50,51]. These principles are important if policy is to provide an appropriate systematic framework for the design and delivery of PHC services, rather than a collection of ad hoc responses to felt needs.

## Conclusion

This study was predicated on (1) the ongoing need to improve poor health outcomes in rural and remote communities through improved access to health services and (2) the belief that the time is ripe to build upon the significant achievements in relation to innovative models of PHC since the first National Rural Health Strategy. Rather than seeking more and more innovation, progress will be made by garnering the knowledge gained since 1993 and enhancing service access through the wider implementation of models that have been shown to be successful.

Unfortunately, despite many descriptive accounts, comprehensive service evaluations have been lacking. As a result, our systematic review of the Australian literature does not reflect a well-established body of knowledge based on rigorous and comprehensive evaluations but rather a preponderance of largely descriptive studies in the published literature. This paucity of evaluations is hardly surprising given a policy environment that has been characterised by a notable absence of a national PHC policy, continual funding of 'innovative' pilots, and a dominant focus on workforce issues, rather than the strategic development of comprehensive models of PHC service delivery.

The systematic review did, however, highlight a number of exemplary models of PHC service delivery which have been evaluated and shown to be successful in meeting their stated goals [20,24,26,31,37,41]. The conceptual framework that emerged from this review provides an important paradigm to underpin future policy development and program funding. The identified 'environmental enablers' assist us to understand what a policy context conducive to positive change might look like. The 'essential service requirements' which characterise the success of these exemplars are amenable to generalisation, adaptation and evaluation in other regions. This is the current policy challenge and currently the subject of further research.

## Competing interests

The authors declare that they have no competing interests.

## Authors' contributions

JW, JSH, RW and PK conceptualised and designed the study. All authors were involved in the literature searches, extraction and analysis of data. JW wrote the first draft and all authors contributed to the final draft.

## Pre-publication history

The pre-publication history for this paper can be accessed here:


